# Depressive symptom trajectories and incident metabolic syndrome in middle-aged and older adults: A longitudinal analysis of the ELSA study

**DOI:** 10.3389/fpsyt.2025.1666316

**Published:** 2025-09-17

**Authors:** Xuhui Chen, Jiaofen Wu, Ying Wang, Yulian He, Honghua Ye, Jianhui Liu

**Affiliations:** ^1^ Department of Pharmacy, Ningbo Medical Center LiHuiLi Hospital, The Affiliated LiHuiLi Hospital of Ningbo University, Ningbo, Zhejiang, China; ^2^ Department of Cardiology, Ningbo Medical Center of Lihuili Hospital, Ningbo, Zhejiang, China

**Keywords:** depressive symptoms, metabolic syndrome, trajectory, ELSA, older adults

## Abstract

**Background:**

The association between late-life depressive symptoms and metabolic syndrome (MetS) remains a critical public health concern, yet most existing evidence relies on cross-sectional designs that fail to capture the dynamic nature of depression. This longitudinal study aimed to investigate how depressive symptom trajectories influence MetS risk in middle-aged and older adults, while examining potential effect modification by sociodemographic and lifestyle factors.

**Methods:**

Using data from the English Longitudinal Study of Ageing (ELSA), we identified three trajectories of depressive symptoms (persistent low, moderate, and high) through group-based trajectory modeling (GBTM) across four survey waves. Multivariable logistic regression assessed associations between trajectories and incident MetS, adjusted for age, sex, education, marital status, smoking, drinking, and income. Stratified analyses evaluated effect modification by these factors.

**Results:**

Participants with persistent moderate (OR=1.08, 95% CI: 1.03–1.15) and high (OR=1.07, 1.01–1.14) trajectories had significantly higher MetS risk versus the low trajectory. Associations were strongest in adults <65 years, married individuals, and those with smoking/drinking habits (p <0.05), but did not vary by sex. Physical activity mediated 18.9% of the total effect (95% CI: 5–37%).

**Conclusion:**

Dynamic depressive symptoms independently predict MetS risk, with amplified effects in younger, married, and health-risk subgroups. Targeted interventions addressing both depressive symptoms and modifiable behaviors (e.g., physical activity) may mitigate metabolic risk in aging populations.

## Introduction

Metabolic syndrome (MetS) is a collection of related metabolic risk factors for cardiovascular disease, such as abdominal obesity, dyslipidemia, impaired glucose tolerance, and hypertension (1). It has emerged as a major global health challenge, with prevalence rates showing a concerning upward trajectory worldwide over the past two decades. Epidemiological data reveal particularly striking patterns across different regions: in Asia-Pacific region, a comprehensive analysis of 15 countries including South Korea demonstrates prevalence rates ranging substantially from 11% to 49% (2), This considerable heterogeneity likely reflects differences in study methodologies, age structures of populations sampled, stages of economic development, and diverse lifestyle and genetic factors across the region. In the UK, approximately one in three adults aged 50 years and older had MetS (3). Meanwhile, in the United States, population studies indicate that more than 30% of adults now meet diagnostic criteria for MetS (4, 5). Previous studies have shown that individuals diagnosed with MetS have a twofold increased risk of cardiovascular events and a fivefold increased risk of type II diabetes (6, 7). The economic burden of MetS-related complications is substantial, with annual costs for cancer, cardiovascular diseases, and obesity-related conditions increasing by 175%, 159%, and 140%, respectively (8). This significant financial impact underscores the urgent need for early intervention to prevent MetS onset and progression. Consequently, numerous studies have investigated risk factors and preventive mechanisms for MetS (9–11).

Depressive symptoms constitute a complex and heterogeneous syndrome characterized by persistent low mood, negative self-evaluation, and psychomotor retardation. Depressive symptoms affect a significant proportion of elderly individuals worldwide, with reported prevalence rates varying from 4.5% to 37.4% in different populations (12, 13). As a major public health concern, depression significantly impairs both physical and mental wellbeing, particularly among middle-aged and older adults (14). Considering its importance to both clinical practice and public health, the association between depressive symptoms in older adults and MetS has emerged as a crucial research topic. Although studies have explored the cross-sectional association between depressive symptoms and MetS (15, 16), research on the longitudinal trajectory patterns of depressive symptoms and MetS risk is still limited. Wu Q et al. (17) studied the association between depressive symptoms and 5-year MetS in elderly Chinese, but only considered baseline depressive status and did not take into account the dynamic changes in depressive symptoms. The 30-year follow-up study by Moorehead et al. (18) confirmed a bidirectional association between depression and metabolic health, but did not examine the dynamic patterns of depressive symptoms. This represents an important knowledge gap, as depression exhibits substantial heterogeneity in its clinical course. Cross-sectional approaches only capture static snapshots of depressive symptoms, failing to account for their dynamic nature. A seminal study (19) tracking 20 participants over seven months revealed significant between-person and within-person variability in symptom patterns, demonstrating that depressive symptoms may persist, fluctuate, or remit over time as individuals transition between depressed and non-depressed states. This temporal variability underscores the limitations of single-timepoint assessments and highlights why repeated measurements of symptom trajectories provide superior predictive validity for understanding their impact on MetS development.

More and more evidence suggests that physical activity may be an important behavioral mechanism linking depressive symptoms to metabolic health. Depressive symptoms often lead to a decrease in physical activity levels (20), while regular physical activity has been shown to improve metabolic indicators through various pathways, including improving endothelial function (21), glucose metabolism (22), reducing systemic oxidized LDL concentration (23), improving mitochondrial quality (24), etc. However, it is currently unclear to what extent physical activity mediates the dynamic changes in depressive symptoms and the association with MetS risk. Therefore, this study will also specifically evaluate the mediating effect of physical activity on the association between depression trajectory and MetS, providing a theoretical basis for developing targeted intervention measures.

To our knowledge, no studies using the English Longitudinal Study of Ageing (ELSA) database have investigated associations between trajectories of depressive symptoms and MetS. This study adopts a group-based trajectory model (GBTM) design, aiming to: (1) identify different longitudinal trajectory patterns of depressive symptoms in middle-aged and elderly populations; (2) analyze the association between these trajectories of depressive symptoms and newly diagnosed MetS; (3) explore the potential modifying effects of sociodemographic and lifestyle factors on this association; (4) evaluate the mediating role of physical activity in the relationship between depression trajectory and MetS. We hope to provide new epidemiological evidence for the relationship between the dynamic changes of depressive symptoms and metabolic health through longitudinal data from the ELSA database.

## Methods

### Data source and study population

This study utilized data from the ELSA, a nationally representative, multidisciplinary cohort study of individuals aged 50 and older residing in England. Since its inception in 2002, ELSA has conducted biennial follow-up surveys, collecting comprehensive data on participants’ economic status, family circumstances, behavioral patterns, social participation, biological markers, retirement status, and health and well-being (25).We analyzed data from four survey waves (2002-2008). From the initial ELSA sample, we excluded participants without follow-up data for the depressive symptom trajectories (n = 13734), those under the age of 50 (n =262), and those with missing data on MetS components (n =3040) as well as individuals with pre-existing metabolic syndrome at baseline (n = 1014). This resulted in a final analytical sample of 1,752 participants.

### Depressive symptoms and MetS

An 8-item version of the Center for Epidemiologic Studies Depression Scale (CES-D) was used to evaluate depressive symptoms (26), which has demonstrated reliability in measuring depressive symptoms among older adults (27). Each item was scored dichotomously (1 point for depressive responses), yielding a total score that spans from 0 to 8, where higher scores indicate more severe depressive symptoms.

In our analysis, depressive symptom trajectories derived from the 2002, 2004, 2006 and 2008 surveys served as the exposure variable, while incident MetS was the outcome variable. MetS was characterized by the National Cholesterol Education Program Adult Treatment Panel III criteria (28), requiring the presence of at least three of the following five components: (1) hypertriglyceridemia (triglycerides ≥1.7 mmol/L); (2) low HDL cholesterol (<1.03 mmol/L in men or <1.29 mmol/L in women); (3) abdominal obesity (waist circumference >102 cm in men or >88 cm in women); (4) elevated blood pressure (systolic BP ≥130 mmHg or diastolic BP ≥85 mmHg, or use of antihypertensive medication, or self-reported hypertension); and (5) elevated fasting glucose (≥5.6 mmol/L or use of antidiabetic medication or self-reported diabetes).

### Other covariates

Several covariates were included in the analysis. Age and sex were recorded for all participants. The classification of educational attainment included below high school, high school, college or above. Participants’ smoking and drinking status was self-reported in response to the questions “Have you ever smoked cigarettes?” and “In the past 12 months, have you taken an alcoholic drink?”. Respondents were categorized as either never smokers or former/current smokers and as either never drinkers or current drinkers. Marital status was grouped into married/partnered, separated/divorced/widowed, or never married. Functional disability was assessed using validated measures of basic and instrumental activities of daily living. The Activities of Daily Living (ADL) scale evaluated six fundamental self-care tasks: feeding, bathing, dressing, transferring (getting in/out of bed), walking across a room, and using the toilet. The Instrumental Activities of Daily Living (IADL) scale assessed five more complex daily living skills: telephone use, financial management, medication administration, grocery shopping, and meal preparation. Participants reporting any degree of difficulty in performing one or more items on either scale were classified as having functional disability. Physical activity levels were assessed and categorized as vigorous (participants engaging in at least one session per week of high-intensity physical activity), moderate (at least one session per week of moderate-intensity activity), or light (less than moderate activity). Income refers to the total household income, including both spouses’ work income, investment income, and government welfare income such as pensions and annuities.

### Statistical analysis

GBTM, a specialized form of finite mixture modeling, was employed to identify distinct subgroups of participants following similar developmental trajectories over time (29). In our analysis, we used the “gbmt” package in R to perform GBTM on continuous CES-D scores from 2002 to 2008, estimating depressive symptom trajectories through censored normal distribution. To determine the optimal number of trajectory groups, we initially evaluated models with 1 to 6 groups. Model selection was based on the Bayesian information criterion (BIC), with additional consideration of group size (minimum 5% of participants) and average posterior probability (minimum 0.7) (29, 30).

Using the persistent low depressive symptom trajectory as reference, we conducted multinomial logistic regression analyses across three progressively adjusted models to examine associations between MetS and depressive symptom trajectories. Model 1: Unadjusted (trajectories of depressive symptoms only). Model 2: Adjusted for demographic confounders (age, sex, education, marital status).Model 3: Additionally adjusted for health behaviors (smoking, alcohol use), income level, and functional disability. The proportional hazards assumption for these logistic regression models was verified using Schoenfeld residuals.

To manage missing covariate data, multiple imputation with chained equations was applied using the ‘mice’ package in R. We generated 10 imputed datasets with a maximum of 50 iterations per imputation, using a fixed random seed (123) for reproducibility. From these 10 imputed datasets, we selected the first complete dataset for our final analysis. This single imputed dataset was then merged with the original complete cases to form the analytical dataset used in all subsequent models.

Continuous variables are expressed as mean ± standard deviation (SD), while categorical variables are shown as frequency (percentage). Baseline characteristics across depressive trajectory groups were compared using one-way ANOVA, Kruskal-Wallis test, or chi-square test as appropriate. R software (version 4.3.2) was used for all analyses, and statistical significance was considered at p < 0.05 (two-tailed).

To investigate whether the association between trajectories of depressive symptoms and MetS varied by demographic characteristics, we conducted stratified analyses to assess potential effect modification by: (1) age (dichotomized at 65 years), (2) sex (male/female), (3) marital status, (4) educational attainment, (5) smoking status, and (6) alcohol consumption. These subgroup analyses were performed by introducing interaction terms between the trajectory groups and each demographic factor in separate multivariable logistic regression models, while maintaining adjustment for all other covariates in the base model.

We conducted a sensitivity analysis restricting to participants aged ≥55 year to assess whether the depression-MetS association was robust to age cut-point selection.

Mediation analysis followed Baron and Kenny’s approach (31) to examine whether baseline physical activity mediated the relationship between trajectories of depressive symptoms and MetS: (1) Linear regression assessed depressive trajectory-MetS association. (2) Linear regression tested physical activity-depressive trajectory association. (3) Linear regression evaluated depressive trajectory-MetS association with physical activity as mediator. At last, we employed nonparametric bootstrapping (1000 resamples) to estimate total, indirect, and direct effects (32).

### Ethical considerations

ELSA adhered rigorously to the ethical guidelines outlined in the Declaration of Helsinki and obtained ethical approval from the London Multicenter Research Ethics Committee for all study waves, ensuring that written informed consent was secured from all participants. Additionally, ethical approval for this study was granted by the Lihuili Hospital Ethics Review Board (KY2025ML037), in compliance with the principles of the Declaration of Helsinki.

## Results

### Depressive symptom trajectories

**Figure 1:**
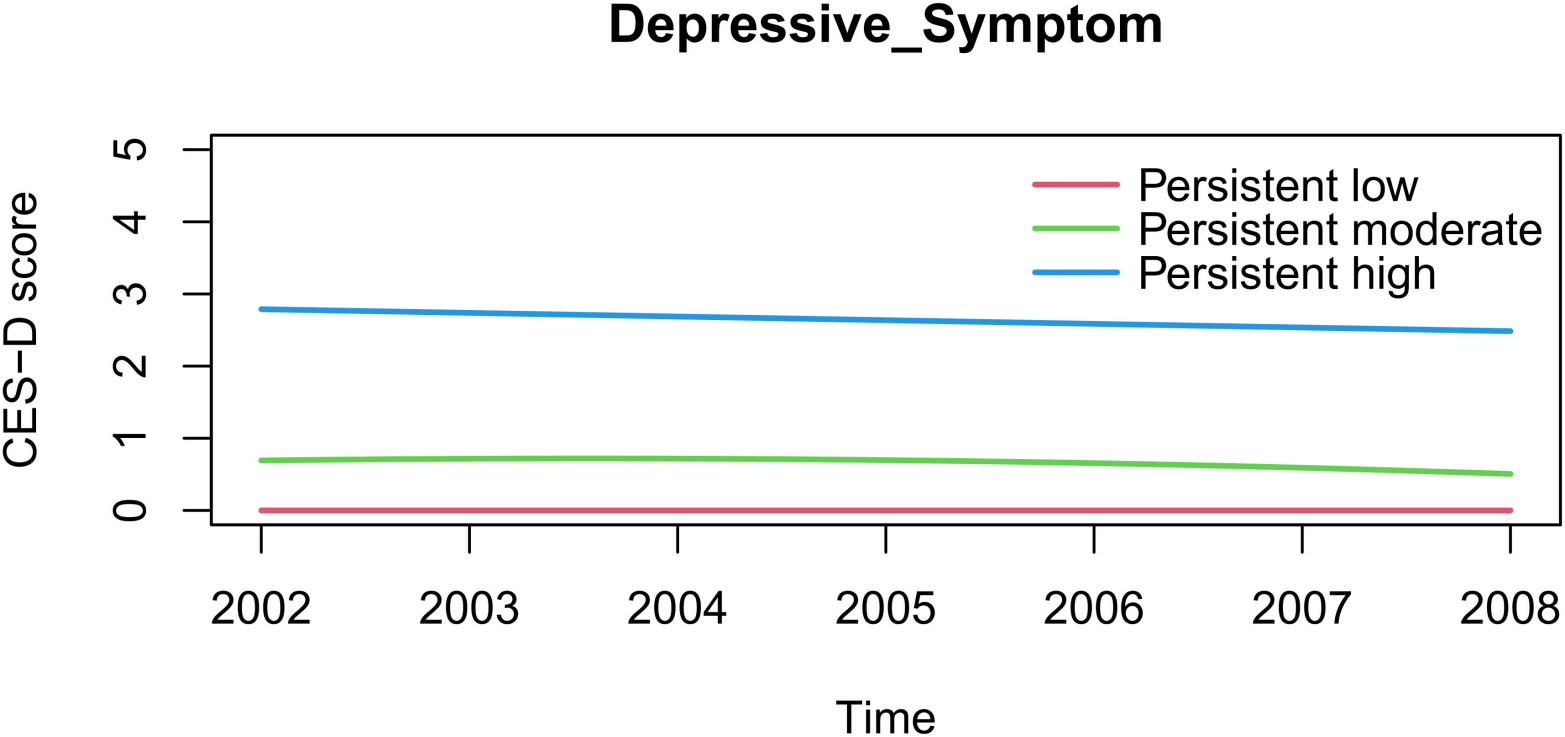
Trajectories of depressive symptoms.

We systematically evaluated model fit indices to determine the optimal number of depressive symptom trajectories (**Table 1**). The three-group solution demonstrated the best balance between model fit and parsimony when compared to more complex models (4–6 groups). This parsimonious model adequately captured population heterogeneity while minimizing overfitting risks and maintaining clinical interpretability. The final trajectories (**Figure 1**) comprised: Persistent low (n=342, 19.52%); Persistent moderate (n=836, 47.72%); Persistent high (n=574, 32.76%). **Table 2** presents maximum likelihood estimates for the three-group model parameters. Each trajectory showed: Distinct intercept and slope parameters; Mean posterior probabilities >0.85; Clear separation with minimal between-group overlap.

**Table 1:** Statistical fitting of depression trajectories in middle-aged and elderly individuals in ESLA.

Fit statistic	Number of classes					
	1	2	3	4	5	6
BIC	27412.85	21615.25	-465672.39	-466030.83	-466052.57	-466115.54
AIC	27392.28	21560.41	-465747.79	-466154.21	-466223.94	-466321.19
Class proportion	Class 1,100%	Class 1,61%	Class 1,20%	Class 1,19%	Class 1,20%	Class 1,20%
		Class 2,39%	Class 2,48%	Class 2,39%	Class 2,41%	Class 2,41%
			Class 3,32%	Class 3,26%	Class 3,21%	Class 3,19%
				Class 4,16%	Class 4,2%	Class 4,2%
					Class 5,16%	Class 5, 2%
						Class 6,16%
APP	Class 1,1.00	Class 1,0.96	Class 1,1.00	Class 1,1.00	Class 1,1.00	Class 1,1.00
		Class 2,0.99	Class 2,0.96	Class 2,0.89	Class 2,0.90	Class 2,0.90
			Class 3,0.96	Class 3,0.85	Class 3,0.85	Class 3,0.85
				Class 4,0.91	Class 4,0.82	Class 4,0.84
					Class 5,0.92	Class 5,0.89
						Class 6,0.92

**Table 2:** The final three trajectory model of ESLA depression score for middle-aged and elderly people.

Trajectory group	Parameter	Maximum likelihood estimates			
		Est	SE	z-value	p-value
Class 1: Persistent low	Intercept	< 0.001	< 0.001	0	1.000
Class 2: Persistent moderate	Intercept	0.647	0.030	21.724	< 0.001
	Linear (t)	0.056	0.018	3.161	0.002
	Quadratic (t²)	-0.011	0.002	-5.037	< 0.001
Class 3: Persistent high	Intercept	2.839	0.053	53.811	< 0.001
	Linear (t)	-0.051	0.012	-4.401	< 0.001

### Participant characteristics

**Table 3** describes the demographic and health characteristics of the 1,752 participants grouped by depressive symptom trajectories at baseline. The median age of participants was 60.8 years, with the majority being female (55.4%) and having college or higher education (47.4%). Most participants were married or partnered (75.3%). Regarding lifestyle factors, 59.8% were former or current smokers, while 92.6% had a history of alcohol consumption. Compared to the “persistent moderate” group, the “persistent high” group had significantly more females (67.8% *vs* 53.8%), lower education levels (38.7% *vs* 50.7% college educated), and higher prevalence of functional disability (22.5% *vs* 6.9%, all p<0.001).

**Table 3:** Basic characteristics of different trajectory groups.

Characteristics	Total	Stratified by trajectory group			P
		Persistent low	Persistent moderate	Persistent high	
Number (%)	1752	342	836	574	
Age [mean (SD)]	60.8 (7.7)	60.7 (7.5)	60.6 (7.5)	61.0 (8.1)	0.716
Sex (%)					<0.001
Female	970 (55.4)	131 (38.3)	450 (53.8)	389 (67.8)	
Male	782 (44.6)	211 (61.7)	386 (46.2)	185 (32.2)	
Education (%)					<0.001
Below high school	528 (30.1)	84 (24.6)	223 (26.7)	221 (38.5)	
High school	394 (22.5)	74 (21.6)	189 (22.6)	131 (22.8)	
College or above	830 (47.4)	184 (53.8)	424 (50.7)	222 (38.7)	
Marital_status (%)					<0.001
Married or partnered	1322 (75.5)	287 (83.9)	660 (78.9)	375 (65.3)	
Never married	81 (4.6)	15 (4.4)	37 (4.4)	29 (5.1)	
Separated/divorced/Widowed	349 (19.9)	40 (11.7)	139 (16.6)	170 (29.6)	
Smoking_status (%)					0.019
Ever smokers	1047 (59.8)	198 (57.9)	479 (57.3)	370 (64.5)	
Never smokers	705 (40.2)	144 (42.1)	357 (42.7)	204 (35.5)	
Drinking_status (%)					0.006
Ever drinkers	1653 (94.3)	328 (95.9)	798 (95.5)	527 (91.8)	
Never drinkers	99 (5.7)	14 (4.1)	38 (4.5)	47 (8.2)	
Functional disability (%)					<0.001
No	1549 (88.4)	326 (95.3)	778 (93.1)	445 (77.5)	
Yes	203 (11.6)	16 (4.7)	58 (6.9)	129 (22.5)	
Income [mean (SD)]	23321.4 (28683.3)	25366.6 (21297.0)	24724.1 (34818.9)	20060.1 (21482.5)	0.004
Physical activity (%)					<0.001
Mild	216 (12.3)	20 (5.8)	80 (9.6)	116 (20.2)	
Moderate	832 (47.5)	159 (46.5)	397 (47.5)	276 (48.1)	
Vigorous	704 (40.2)	163 (47.7)	359 (42.9)	182 (31.7)	

### Association between depressive symptom trajectories and MetS

**Table 4** displays the longitudinal associations between depressive symptom trajectories and incident MetS. In the fully adjusted Model 3 (which controlled for age, sex, marital status, educational attainment, smoking and alcohol consumption behaviors, functional disability status and income), compared to the persistent low depressive symptom group (reference category), the persistent moderate depressive symptom group showed a significantly higher risk of developing MetS (OR=1.09, 95% CI: 1.03-1.15, p=0.002). Similarly, the persistent high depressive symptom group also demonstrated an elevated risk (OR=1.07, 95% CI: 1.01-1.14, p=0.019).

**Table 4:** Multivariate logistic regression analysis of depression trajectory and MetS.

Trajectory group	Model 1		Model 2		Model 3	
	OR (95% CI)	P	OR (95% CI)	P	OR (95% CI)	P
Persistent low	1.00 (reference)		1.00 (reference)		1.00 (reference)	
Persistent moderate	1.09(1.04-1.15)	0.001	1.09(1.04-1.15)	0.001	1.09(1.03-1.15)	0.002
Persistent high	1.11(1.05-1.17)	<0.001	1.10(1.03-1.16)	0.002	1.07(1.01-1.14)	0.019

Model 1 was unadjusted.

Model 2 additionally adjusts for sociodemographics (age and sex, education, marital status).

Model 3 additionally adjusts for health behaviors (smoking, alcohol use), income and functional disability.

### Subgroup analysis of depressive symptom trajectories and MetS

**Figure 2:**
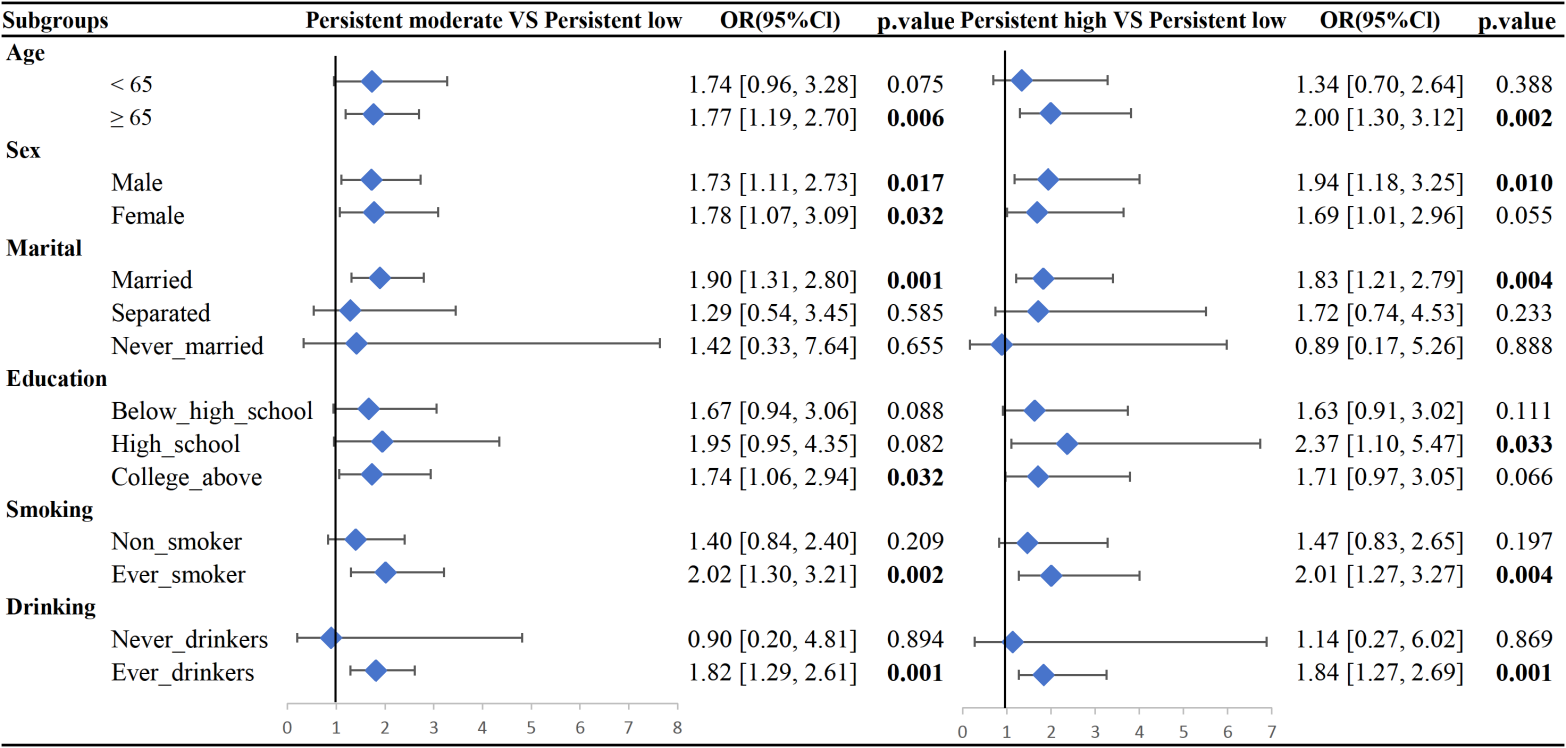
Association between depression trajectories and metabolic syndrome, stratified by age group, sex, marital status, education, smoking status, and drinking status.

The subgroup analysis (**Figure 2**) showed that among individuals younger than 65 years, neither the persistent moderate nor persistent high depressive symptom groups demonstrated statistically significant associations with MetS compared to the persistent low group. In contrast, among those aged 65 years or older, both the persistent moderate (OR=1.77, 95% CI: 1.19–2.70, p = 0.006) and persistent high (OR=2.00, 95% CI: 1.30–3.12, p = 0.002) groups were significantly associated with MetS. In males, both the persistent moderate (OR=1.73, 95% CI: 1.11–2.73, p = 0.017) and persistent high (OR=1.94, 95% CI: 1.18–3.25, p = 0.010) groups showed significant associations with MetS, while in females, the persistent moderate group was significantly associated (OR=1.78, 95% CI: 1.07–3.09, p = 0.032), and the persistent high group showed a borderline association (OR=1.69, 95% CI: 1.01–2.96, p = 0.055), with slightly weaker effect sizes than in males. Among ever-smokers, both the persistent moderate (OR=2.02, 95% CI: 1.30–3.21, p = 0.002) and persistent high (OR=2.01, 95% CI: 1.27–3.27, p = 0.004) groups were significantly associated with MetS, whereas no significant associations were observed in never-smokers (p > 0.05). Similarly, among ever-drinkers, both the persistent moderate (OR=1.82, 95% CI: 1.29–2.61, p = 0.001) and persistent high (OR=1.84, 95% CI: 1.27–2.69, p = 0.001) groups showed significant associations with MetS, while no significant associations were found in never-drinkers (p > 0.05).

### Mediation analysis

**Figure 3:**
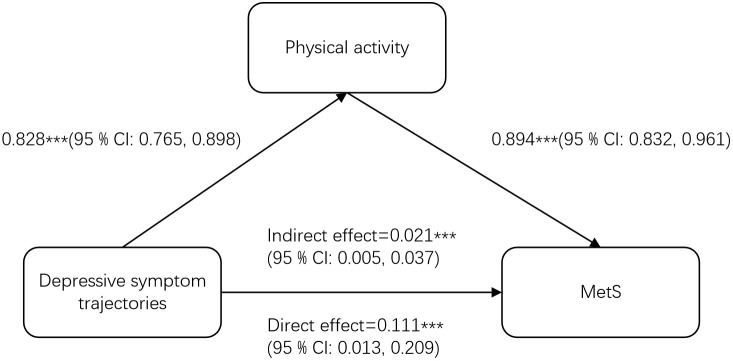
Mediation effect.

As shown in **Supplementary Table S1**, when physical activity was included as a mediator in Model 4, the persistent moderate depressive trajectory group remained significantly associated with MetS (OR=1.08, 95% CI [1.03, 1.14], p = 0.002), while the persistent high depressive trajectory group also showed significant association (OR=1.06, 95% CI [1.00, 1.13], p = 0.039), indicating that physical activity partially mediated the association between trajectories of depressive symptoms and MetS. Bootstrap analysis revealed that the total effect of trajectories of depressive symptoms on MetS was 0.111 (95% CI: 0.013, 0.209), with an indirect mediation effect through physical activity of 0.021 (95% CI: 0.005, 0.037). These results demonstrate that physical activity served as a significant mediator in the association between trajectories of depressive symptoms and MetS, accounting for 18.92% of the total effect. The mediation pathway model is illustrated in **Figure 3**.

### Sensitivity analyses

The sensitivity analysis is consistent with the main analysis results. When the age is set to ≥ 55 years, after adjusting for age, gender, marital status, education level, smoking and drinking behavior, and functional loss, individuals with persistent moderate depressive symptoms have a higher risk of developing MetS compared to individuals without depressive symptoms (**Supplementary Table S2**). Subgroup analysis shows that among individuals under 65 years, married, with a high school education, smoking, and drinking, there is a significant correlation between moderate to high levels of depression and metabolic syndrome (**Supplementary Figure S1**).

## Discussion

This longitudinal study examined the association between depressive symptom trajectories and MetS in middle-aged and older adults. Through four survey waves, we identified three distinct depressive symptom trajectories (persistent low, moderate, and high), finding that individuals with moderate and high trajectories of depressive symptoms had significantly higher MetS risk compared to those with low trajectories. Subgroup analyses revealed these associations varied by age, marital status, and lifestyle factors (smoking, alcohol use) but not by gender. Our findings of three trajectories differ from another ELSA study (33) that identified five trajectories, likely due to their inclusion of “Health and Retirement Study” participants and use of predefined depressive score cutoffs rather than our data-driven GBTM approach.

The observed association between depressive symptoms and incident MetS aligns with previous cross-sectional studies (34, 35). A 2018 meta-analysis of 12 studies (36) similarly supported this relationship, with all three longitudinal and seven of nine cross-sectional studies reporting positive associations. However, inconsistencies exist in the literature, with some studies reporting null associations. Including a Norwegian study in 60–89 year-olds (37), a U.S. study finding associations only in White persons (38), and a Japanese study showing only depression-fasting hyperglycemia links (39). These discrepancies may stem from methodological differences (study designs, assessment tools, diagnostic criteria) and population variations (cultural, lifestyle, demographic).

The association between depressive symptoms and MetS involves complex pathophysiological mechanisms that have been elucidated through several lines of research. Individuals experiencing depressive symptoms often engage in unhealthy lifestyle choices, such as smoking, excessive alcohol consumption, poor sleep quality, reduced physical activity, and a diet high in fats and carbohydrates. These behavioral risk factors can contribute to the deterioration of cardiovascular health and increase the risk of developing MetS (40). Furthermore, patients with depression commonly exhibit pathological and physiological characteristics indicative of dysregulation within their homeostatic systems. These features are closely linked to the onset of MetS. The overactivation of the hypothalamic-pituitary-adrenal (HPA) axis results in excessive secretion of glucocorticoids, which promotes lipid storage and fat production, leading to abdominal fat accumulation, elevated blood glucose and triglyceride levels, and ultimately MetS (41–43). Additionally, the activation of inflammatory responses facilitates the release of lipids into the bloodstream, resulting in a decrease in high-density lipoprotein cholesterol and phospholipids, while increasing triglyceride levels (44). Prolonged activation of the HPA axis and inflammatory processes may impair insulin sensitivity and directly impact pancreatic beta cells, thereby disrupting glucose metabolism (45, 46). Moreover, the dynamic imbalance between the sympathetic and parasympathetic nervous systems can enhance nervous system excitability, elevate catecholamine hormone levels, and raise blood pressure, blood glucose, and lipid levels, all of which contribute to insulin resistance and the development of MetS (20). In summary, there exists a bidirectional interaction between various internal homeostatic systems and both mental and physical health, with dysregulation of these systems potentially leading to the comorbidity of depression and MetS.

Our findings identify smoking and alcohol consumption as significant risk factors for MetS in depressed individuals, consistent with previous reports. Jinhee Lee’s study (47) demonstrated that depressed male heavy drinkers had a 2.75-fold increased risk of MetS compared to non-depressed males. The pathophysiological basis for this association includes multiple mechanisms: smoking and alcohol consumption elevate blood pressure (48), depression is associated with poorer health behaviors including smoking, unhealthy diet, and physical inactivity (49), and frequent alcohol intake is linked to impaired fasting glucose, abdominal obesity, and hypertriglyceridemia in men (50). Furthermore, alcohol may stimulate appetite and promote overeating, thereby increasing obesity risk (51).

Moreover, our results revealed that although women were more likely to exhibit a high depressive symptom trajectory, the associated risk of incident MetS did not significantly differ between genders within the same trajectory group. This suggests that the metabolic consequences of sustained depressive symptoms may be similar across genders, even if the underlying prevalence of depression differs. This result is consistent with several previous studies reporting a significant association between depression and MetS in both men and women (52, 53). However, it appears to contradict other reports that found stronger associations in women (54, 55). We propose that these discrepancies may be partly explained by age differences across study populations. Gender differences in depression incidence typically emerge during adolescence and persist through midlife, with the gap being most pronounced before age 50 (56, 57). Our study specifically included participants aged ≥50 years, whereas many studies reporting gender-specific effects included younger adults (e.g., from age 20). Thus, the hormonal and psychosocial changes associated with mid- and late-life may help explain the similar depression–MetS risk we observed in older men and women.

Furthermore, our analysis identified marital status as a significant effect modifier in the association between depressive symptom trajectories and MetS, with married individuals exhibiting a stronger association. This finding is supported by several previous studies conducted across diverse populations. For example, research by Ajlouni et al. (58) indicated that married individuals in Jordan had more than twice the risk of developing MetS compared to their single counterparts (OR=2.26). Similarly, Latha et al. (59) reported marriage as a significant risk factor for MetS in southern India (PR=2.243), and Sirdah et al. (60) observed a notable correlation between marital status and MetS among Palestinian adults in the Gaza Strip, identifying marriage as a potential trigger for MetS development. However, contradictory evidence also exists. Some studies suggest that being unmarried—particularly single status—is associated with a higher risk of MetS. For instance, Woldekidan et al. (61) proposed that single men may adopt less healthy lifestyles and face increased cardiovascular risks, contributing to MetS development. Moorthy et al. (62) attributed a protective effect of marriage to the social support and stability it provides, which may promote healthier behaviors related to diet, physical activity, and stress management. We propose that these conflicting conclusions may arise from the fact that most studies consider marital status as a binary variable without accounting for marital quality. It is plausible that high-quality marriages confer protective benefits, whereas strained marital relationships may exacerbate health risks, including MetS—particularly in the context of pre-existing depression (63). Unfortunately, our dataset did not include direct measures of marital quality, which limits further exploration of this mechanism. Future studies should incorporate detailed assessments of relationship satisfaction, social support, and marital stress to elucidate how specific marital contexts modify metabolic risk in individuals with depression.

A key finding of our study is the mediating role of physical activity in the depression-MetS association. Depression frequently leads to reduced physical activity (64), while exercise benefits multiple metabolic parameters through various mechanisms: enhancing glucose uptake via GLUT4 translocation (65), improving mitochondrial function in skeletal muscle (66), reducing abdominal adiposity (67, 68), increasing insulin sensitivity (69, 70), improving vascular function (71, 72), and regulating metabolic gene expression (73, 74). These findings suggest that physical inactivity may represent a critical pathway through which depressive symptoms increase MetS risk. Research has demonstrated that various forms of physical activity—including walking, yoga, qigong, resistance training, and tai chi can effectively alleviate depressive symptoms in older adults (75). Furthermore, emerging evidence suggests that combined aerobic and resistance exercise may yield superior benefits for mitigating MetS compared to either modality alone (65).

To our knowledge, this is the first study to use the ELSA database to investigate the relationship between depression trajectories and MetS, and to discuss the mediating role of social activity. Our research has several limitations that need to be considered. Firstly, this study did not include information on the use of antidepressants. Antidepressant drug data was only available during the ELSA wave in 2004 and 2008, which hindered its consistent inclusion as a time-varying covariate throughout the entire modeling period (2002-2008).Due to the increased risk of developing MetS in individuals with the use of antidepressants (76), if participants with severe depressive symptoms use these medications more frequently, the direct effect of depression trajectory on MetS may be overestimated. Future studies with comprehensive longitudinal medication records are warranted to confirm our findings. Secondly, physical activity data relies on self-reported questionnaires, which can easily overestimate actual activity levels and do not differentiate between activity types (such as aerobic *vs*. resistance exercise). Future research can further differentiate between activity types and implement targeted interventions to prevent the occurrence of MetS. Third, although this study adjusted for a range of important sociodemographic and behavioral covariates, residual confounding may persist due to unmeasured or imprecisely measured variables. Metabolic syndrome is a multifactorial condition influenced by a complex interplay of genetic, lifestyle, and environmental factors. While we accounted for key factors like smoking, alcohol use, and physical activity intensity, we lacked detailed data on other potential confounders such as specific dietary patterns, genetic predisposition, and environmental determinants, all of which are known to influence metabolic health. Furthermore, although we adjusted for income and education, socioeconomic status is a multifaceted construct that may not be fully captured by these metrics. Additionally, factors such as living alone (as an indicator of social isolation) and history of clinical depression, which may influence both depressive trajectories and MetS, were not included as covariates. While these are important, their inclusion could lead to over-adjustment for variables that lie on the hypothesized causal pathway or are closely related to the exposure. For instance, a clinical depression history is a strong predictor of current depressive symptoms and adjusting for it might attenuate the very association we aim to study. The absence of these and other variables means we cannot rule out their potential role in the observed associations between depressive symptom trajectories and MetS. Future studies incorporating more precise and comprehensive measurements of these covariates would be valuable to confirm our findings. Finally, this study used the CES-D scale as a depression assessment tool. Although the scale is easy to operate, it cannot distinguish depression subtypes, and different subtypes may affect metabolism through different mechanisms. Future research should consider distinguishing subtypes of depression and studying the impact of non depression subtypes on MetS.

In conclusion, our findings demonstrate that trajectories of depressive symptoms in middle-aged and older English adults directly increase MetS risk while also exerting indirect effects through reduced physical activity. These results suggest that interventions targeting both depressive symptom management and lifestyle modifications, particularly physical activity promotion, may help mitigate MetS risk in this population.

## Data Availability

The original contributions presented in the study are included in the article/**Supplementary Material**. Further inquiries can be directed to the corresponding authors.
