# Supplementary material for: Depressive symptom trajectories and incident metabolic syndrome in middle-aged and older adults: A longitudinal analysis of the ELSA study
Source: Front Psychiatry. 2025 Sep 17;16:1666316. doi: 10.3389/fpsyt.2025.1666316 (PMC12483997; doi:10.3389/fpsyt.2025.1666316)
Supplement: Supplementary file 1 [file Table1.docx]

Table S 1 Multivariate logistic regression analysis of depression trajectory and metabolic syndrome when physical activity serves as a mediator

|  | OR (95% CI) | p value |
| --- | --- | --- |
| Persistent low | 1·00 (reference) |  |
| Persistent moderate | 1.08(1.03-1.14) | 0.002 |
| Persistent high | 1.06(1.00-1.13) | 0.039 |

Table S2 Multivariate logistic regression analysis of depression trajectory and MetS

|  | Model 1 | | Model 2 | | Model 3 | |
| --- | --- | --- | --- | --- | --- | --- |
|  | OR (95% CI) | P | OR (95% CI) | p | OR (95% CI) | p |
| Persistent low | 1.00 (reference) |  | 1.00 (reference) |  | 1.00 (reference) |  |
| Persistent moderate | 1.12(1.05-1.19) | <0.001 | 1.12(1.05-1.19) | <0.001 | 1.11(1.04-1.18) | <0.001 |
| Persistent high | 1.11(1.04-1.18) | <0.001 | 1.10(1.02-1.17) | <0.001 | 1.07(1.00-1.14) | 0.062 |

Model 1 was unadjusted.
Model 2 additionally adjusts for sociodemographics (age and sex, education, marital status).
Model 3 additionally adjusts for health behaviors (smoking, alcohol use), income and functional disability.


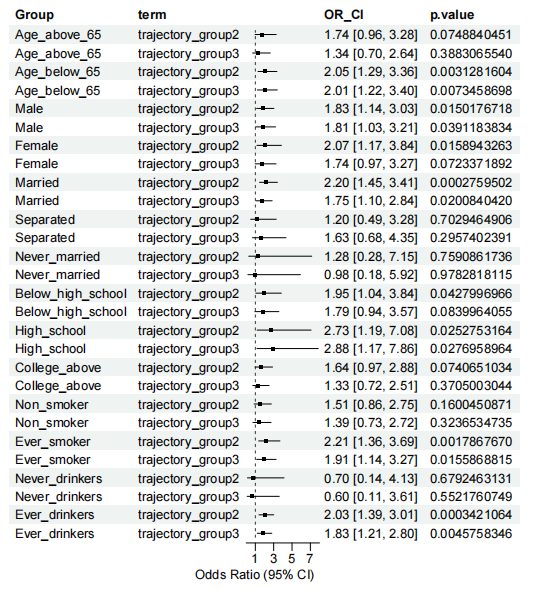


Figure S1 Association between depression trajectories and metabolic syndrome, stratified by age group, sex, marital status, education, smoking status, and drinking status
